# OPTImising the implementation of pulMonary rehAbiLitation in people with chronic obstructive pulmonary disease (the OPTIMAL study): mixed methods study protocol

**DOI:** 10.1186/s12890-020-01322-4

**Published:** 2020-11-02

**Authors:** Sarah Hug, Vinicius Cavalheri, Daniel F. Gucciardi, Richard Norman, Kylie Hill

**Affiliations:** 1 School of Physiotherapy and Exercise Science, Faculty of Health Sciences, Curtin University, Perth, Australia; 2 Physiotherapy Department, Royal Perth Hospital, Perth, Australia; 3 Allied Health, South Metropolitan Health Service, Perth, Australia; 4 Institute for Respiratory Health, Perth, Australia; 5 School of Public Health, Faculty of Health Science, Curtin University, Perth, Australia

**Keywords:** Chronic obstructive pulmonary disease, Pulmonary rehabilitation, Referral, Uptake, Attendance, Adherence, Co-creation, Participatory research

## Abstract

**Background:**

Chronic obstructive pulmonary disease (COPD) is a common respiratory condition characterised by dyspnoea during daily life. As the disease progresses, people with COPD can experience poor quality of life, reduced exercise capacity, worsening of symptoms and increased hospital admissions. Pulmonary rehabilitation, which includes exercise training, optimises both psychological and physical function, reduces symptoms and mitigates healthcare utilisation in people with COPD. There is, however, a gap in implementation of pulmonary rehabilitation programs, with global access limited to a small fraction of people with COPD. The overall aim of this study is to gather evidence that will optimise the implementation of pulmonary rehabilitation in people with COPD living in Perth, Western Australia.

**Methods:**

This is a mixed methods study protocol informed by a critical realist perspective. The study will comprise four phases. In Phase 1, we will quantify target behaviours of healthcare professionals and people with COPD which are related to the implementation of pulmonary rehabilitation at three tertiary hospitals. In Phase 2, we will conduct semi-structured interviews to explore the determinants of these target behaviours from the perspectives of healthcare professionals, people with COPD and their primary support person. In Phase 3, knowledge gained in Phases 1 and 2 will be used by healthcare professionals and people with COPD to co-create, field test and apply strategies that optimise these target behaviours. In Phase 4, we will re-quantify these target behaviours to determine the influence of co-created strategies. The cost effectiveness of implementing the co-created strategies will be explored by an economic analysis.

**Discussion:**

Understanding current clinical practice and the determinants of target behaviours pertaining to the implementation of pulmonary rehabilitation is crucial when developing strategies that successfully bridge the pulmonary rehabilitation implementation gap. If co-created strategies are effective, more people with COPD living in Perth, Western Australia will have access to pulmonary rehabilitation enabling them to derive the health benefits associated with this intervention.

## Background

Chronic obstructive pulmonary disease (COPD) is a common progressive lung condition characterised by dyspnoea during daily life [1]. In 2017, COPD was the most prevalent chronic respiratory disease globally, with an overall prevalence of 9.8% [2]. Across Australia, approximately 1 in 13 people over the age of 40 have COPD of at least moderate severity [3, 4]. In 2015, COPD was the third highest cause of total disease burden in Australia, costing the healthcare system AU$976.9 million from exacerbations, hospitalisations and medications [5, 6]. As the disease progresses, people with COPD commonly report a deterioration in health-related quality of life, exercise capacity and increased symptoms such as dyspnoea and fatigue during daily life [1, 7]. They are also at an increased risk of experiencing an exacerbation requiring hospitalisation [1, 7]. Pulmonary rehabilitation (PR), which includes exercise training, is an important component in the management of people with COPD [8]. It is well established that in people with stable COPD, PR leads to clinically meaningful improvements in health-related quality of life and exercise capacity, and reduces symptoms such as dyspnoea and fatigue during daily life [9, 10]. There is evidence of a similar effect on these outcomes when exercise training is initiated during or immediately following an exacerbation of the disease [11]. With regard to the healthcare system, PR can reduce healthcare utilisation in both people with stable COPD, and following an exacerbation [11, 12].

Clinical guidelines for the management of people with COPD recommend that PR should be provided to people with stable COPD who experience symptoms during daily life, as well as during or soon after an exacerbation of the disease [7, 8]. Despite guideline recommendations and robust evidence to support its effectiveness [9], PR is underused [13]. In 2015, an international study which included data from Australia, Canada, Ireland, New Zealand, Sweden, the United Kingdom and the United States demonstrated that the proportion of people living with COPD who have access to PR is < 1.2% [14]. Underuse of PR is recognised as an international problem prompting the European Respiratory Society (ERS) and American Thoracic Society (ATS) to call for action to bridge the gap between the documented benefits of PR and implementation issues [13]. In Australia, observational data has shown that nearly half of all people with stable COPD and nearly three-quarters of those following an exacerbation of the disease who may benefit from PR are not referred to PR [15, 16]. Healthcare professionals (e.g. Physicians, General Practitioner’s, Physiotherapists) report barriers to PR referral such as limited knowledge of its benefits, uncertainty around program details (i.e. referral processes, location, content and duration of the program), and limited time to discuss and refer to PR [17–19]. The failure to discuss and advocate for PR in the in-patient and out-patient setting leaves people with COPD largely unaware of both the existence of PR programs and its health benefits. Of equal concern are data showing that of those referred to PR, approximately one third do not attend their initial assessment or commence the program [20, 21]. Reasons provided by people with COPD for not attending an initial assessment for PR or choosing not to commence supervised exercise classes relate their perception of limited benefit of such programs and the claim that they are ‘already completing their own exercise’. [19, 22–24] Following an exacerbation of the disease, feelings of low self-worth and being ‘too unwell’ to exercise also reduce the willingness to accept a referral, attend an initial assessment, commence and adhere to an exercise program [25, 26]. Once people with COPD commence PR, the challenge becomes adherence to the program; however, non-completion rates vary internationally. Observational data from Australia, for example, have shown that people with stable COPD who commence PR attend most sessions [16], suggesting that engagement in PR (through referral and attendance to an initial assessment) is the most crucial hurdle to overcome. Earlier work mapping the PR pathway in people with COPD has used retrospective audits of medical records or simple questionnaires over a short period of time [16, 20, 27, 28]. These studies offer an incomplete picture of the PR pathway and barriers to PR. There is a need to prospectively map the full PR pathway in people with COPD, and acquire a comprehensive understanding of the complexities of behaviours which drive the implementation of PR programs, from the perspectives of both healthcare professionals and people with COPD.

The overall aim of this mixed methods study is to gather evidence that will optimise the implementation of PR in people with COPD living in Perth, Western Australia (WA). The research questions to be answered are as follows:

1. In people with COPD, what is current practice, quantified as specific target behaviours pertaining to the implementation of PR at three tertiary hospitals?
2. From the perspectives of healthcare professionals, people with COPD and their primary support person, what are the barriers and facilitators to the target behaviours pertaining to the implementation of PR?
3. What strategies can be co-created by healthcare professionals and people with COPD with the goal of optimising target behaviours pertaining to the implementation of PR in people with COPD?
4. What is the influence of co-created strategies (from research question 3) on the target behaviours pertaining to the implementation of PR at three tertiary hospitals?

## Methods/design

### Overview

**Fig. 1:**
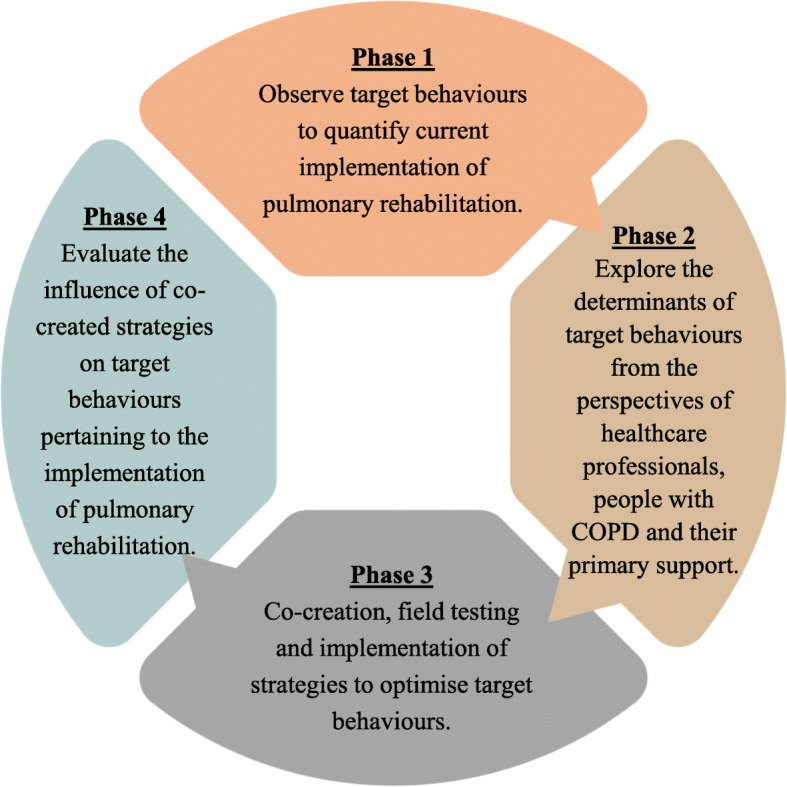
Study design flow diagram

This project will be a 2-year mixed method, co-creation research study undertaken across Fiona Stanley Hospital (FSH), Royal Perth Hospital (RPH) and Sir Charles Gairdner Hospital (SCGH) in Perth, WA. The project has been approved by the Human Research Ethics Committee for the South Metropolitan Health Service (RGS0000003704) with reciprocal approval obtained from the Human Research Ethics Committees for Royal Perth Hospital, Sir Charles Gairdner Hospital, and Curtin University (HRE2020–0095). The research questions will be answered across four consecutive five-month phases (see Fig. 1).

Data collection for this project commenced in August 2020, and will continue until April 2022. A steering committee has been established which comprises the research group and clinicians from all three sites. The committee meets monthly via teleconference to discuss conduct of this study and ensures fidelity during data collection.

### Philosophical positioning

Knowledge of our philosophical perspective is essential for appreciating our construction of knowledge (methodology). The design of this project is informed by a critical realist perspective in which ontology (the nature of reality) and epistemology (the nature of knowledge) are considered as two distinct, separate entities. A key feature of a critical realist perspective is that reality exists independently of human awareness [29]. Observation can increase one’s confidence in the existence of reality, but observation does not dictate it. Reality from a critical realist perspective sees the world divided into the real (co-occurrence of causal mechanisms that may influence events and experiences), actual (events and experiences caused by causal mechanisms) and empirical (observable events or actual experiences) domains [30]. Critical realism seeks to develop *empirically supported explanations* of phenomena via questions of how, why, and under what conditions [31]. It encourages researchers to delve beyond empirical observations to understand what causes particular events and behaviours; acknowledging that some claims may be better approximations of reality than others, and recognising that knowledge of these realities is socially produced, temporally transient, and fallible in nature because of the separation of ontology and epistemology [32]. Critical realism rejects social constructivism notions that knowledge is constructed through interaction with others; rather, it acknowledges that we have individual experiences and there are different perspectives on reality [33]. The separation of ontology and epistemology seen within critical realism allows for methodological plurality. Consistent with the notion of ‘fit for purpose’ [29], our methodological approach involves both quantitative and interdisciplinary qualitative methods to generate insight on the interrelationships between context, mechanisms, and outcomes across four phases. Phases 1 and 2 will enable us to construct knowledge through the three layers of reality. Phase 1 contributes to the *actual* layer of reality via quantification of current clinical practice of target behaviours (see Table 1) pertaining to the implementation of PR in Perth. Phase 1 also contributes to the *real* layer of reality via the identification of factors such as gender, current smoking status, and social support which activate causal mechanisms and therefore may influence the *actual* reality observed across Phase 1. Phase 2 contributes to the *empirical* layer of reality where we will explore experiences of healthcare professionals and people with COPD to understand their capability, opportunity, and motivation to fulfil target behaviours (outlined in Table 1). In Phase 3, knowledge constructed through these three layers of reality will be used as a basis to co-create strategies that optimise how PR is implemented. Phase 4 will see clinical practice of PR implementation re-quantified, with potential to demonstrate change in the *actual* layer of reality with co-created strategies in place.

**Table 1:** Target behaviours to be quantified in Phase 1 and 4

	Adults hospitalised with an exacerbation of COPD	Adults with COPD who attend Respiratory Medicine out-patients
**Referral to PR**	Proportion referred to PR prior to or within 2 weeks of hospital discharge.	Proportion referred to PR within 2 weeks of attendance at a Respiratory Medicine clinic appointment.
**Attendance to an initial appointment**	Proportion who attend an initial assessment with a Physiotherapist to determine suitability to enrol into an exercise training program.	Proportion who attend an initial assessment with a Physiotherapist to determine suitability to enrol into an exercise training program.
**Commence PR**	Proportion who attend at least one exercise training session overseen by a Physiotherapist.	Proportion who attend at least one exercise training session overseen by a Physiotherapist.
**Attendance to PR**	Proportion who attend ≥80% of the scheduled exercise training sessions overseen by a Physiotherapist.	Proportion who attend ≥80% of the scheduled exercise training sessions overseen by a Physiotherapist.
**Provision of a maintenance strategy**	Proportion who are referred to a maintenance program on completion of exercise training sessions overseen by a Physiotherapist.	Proportion who are referred to a maintenance program on completion of exercise training sessions overseen by a Physiotherapist.

*Abbreviations*: *COPD* chronic obstructive pulmonary disease, *PR* pulmonary rehabilitation

### Phase 1: observing target behaviours of healthcare professionals and people with COPD to quantify current implementation of PR

The aim of Phase 1 is to record target behaviours of healthcare professionals and people with COPD to quantify current implementation of PR in people with COPD. This phase will recruit people with COPD who are currently hospitalised with an exacerbation of their condition (i.e. in-patients) as well as people with COPD who are attending Respiratory Medicine out-patient clinics (i.e. out-patients) at any of the three tertiary hospitals. The target behaviours to be quantified are: referral to PR, attendance at an initial assessment, commencement of supervised exercise training classes, adherence to supervised exercise training and provision of a maintenance strategy on program completion (see Table 1).

#### Inclusion criteria

The inclusion criterion for in-patients will be people admitted to one of the three tertiary hospitals with a primary diagnosis of an exacerbation of their COPD. Exclusion criteria are as follows: (i) admitted to the intensive care unit at time of recruitment (ii) unable to ambulate independently and safely, (iii) have cognitive impairment or an inability to understand English, (iv) living in supported residential care prior to admission and/or (v) not expected to survive the admission (i.e. deemed terminal). Cases will be found by undertaking a daily screen of admissions to specific wards across the three tertiary hospitals using the hospital electronic data system.

The inclusion criterion for out-patients will be people who meet the spirometric criteria for COPD (i.e. post bronchodilator forced expiratory volume in 1 s / forced vital capacity [FEV_1_/FVC] < 0.7) [1]. Exclusion criteria include: (i) completed PR within the previous 12 month period, (ii) attend maintenance PR classes within the previous 8 week period, (ii) unable to ambulate independently and safely, (iii) have cognitive impairment or an inability to understand English, (iv) living in supported residential care and/or (v) have an expected survival of less than 6 months. Cases will be found by daily screening Respiratory Medicine clinic lists at the three tertiary hospitals.

#### Informed consent and data collection

Each person who meets the study criteria will be approached and invited to provide written informed consent to participate in Phase 1. Thereafter, the journey of each participant will be tracked with their data collected using Research Electronic Data Capture (REDCap) data collection tools [34] regarding: (i) referral to PR, (ii) attendance to initial assessment (or reason for non-attendance), (iii) commencement of supervised exercise training (or reason for non-commencement), (iv) adherence to PR (or reasons for non-adherence) and (v) provision of a maintenance strategy on PR completion (see Table 1). Researchers and research assistants will prospectively extract these data; (i) using the participant’s medical records, (ii) via discussion with the participant (either in person or via telephone call) and, (iii) via discussion with the treating Physiotherapist. Research assistants involved in data collection have been provided with standardised training and resources, and meet weekly via teleconference ensure fidelity during data collection. The REDCap data collection tools and resources for research assistants can be found on our Open Science Framework website [35].

### Phase 2: exploring the determinants of target behaviours from the perspectives of healthcare professionals, people with COPD and their primary support person

The aim of Phase 2 is to explore the determinants of behaviours related to referral to PR, initial appointment attendance, commencement of PR, adherence to PR and provision of a maintenance strategy on PR completion. Semi-structured interviews will be conducted to gain perspectives of healthcare professionals (across all three sites), people with COPD and where possible their nominated primary support person.

#### Informed consent

During the process of obtaining informed consent for Phase 1, participant’s interest in engaging in Phase 2 and/or Phase 3 of the project will be ascertained. Expression of interest to participate in Phase 2 will be sought via email from healthcare professionals (e.g. Physicians, Nursing, Physiotherapy and other Allied Health Professionals) who are involved in the management of people with COPD. Healthcare professionals, people with COPD and, where possible, their primary support person will be recruited using purposeful sampling and approached for written informed consent to participate in Phase 2. Purposeful sampling ensures a diverse representation of people with COPD (e.g. smokers vs. non-smokers, have vs. have not attended PR, few vs. multiple co-morbid conditions, have vs. have not had been hospitalised for an exacerbation of COPD within the previous 12 months) and healthcare professionals (e.g. Medical, Nursing, Allied health).

#### Interview structure

A behavioural analysis will be conducted to understand the barriers and facilitators to fulfilling the target behaviours pertaining to PR implementation from the perspectives of healthcare professionals, people with COPD and their primary support person. All interviewees will be asked to comment on their capability, opportunity, and motivation [36] to achieve the target behaviours related to PR implementation. Interview questions to explore specific barriers and enablers will be iteratively developed from Phase 1, and will be based on the Theoretical Domains Framework [37], which is a comprehensive framework for the identification and description of factors that influence behaviour. Data-prompting techniques, such as sharing the results of Phase 1 and photos of adults hospitalised with an exacerbation of COPD (from the internet), will be used to probe barriers and facilitators of PR target behaviours. Interviews will be recorded and transcribed verbatim.

### Phase 3: co-creation, field testing and application of strategies to optimise target behaviours pertaining to PR implementation

The aim of Phase 3 is to use knowledge gained across Phase 1 and 2 to co-create, field test and apply strategies to optimise target behaviours pertaining to PR implementation across Perth, WA. Phase 3 will involve collaboration between healthcare professionals and people with COPD (i.e. the co-creators or co-researchers) as they engage in a series of consumer-driven workshops over a 5-month period [38].

#### Informed consent

Participants from Phase 1 who expressed interest in participating in Phase 3 will be recruited using purposeful sampling and approached for written informed consent to participate in this phase. Purposeful sampling ensures the co-creators are a diverse representation of the wider population of healthcare professionals and people with COPD, and will allow us to ensure that power-imbalances or conflicts of interest among participants are avoided.

#### Workshop content

Each workshop will be attended by a researcher, scribe, healthcare professionals who are currently involved in the management of people with COPD and people with COPD (with or without their primary support person). Participatory workshops will commence with a framing workshop during which data will be shared from Phases 1 and 2 to upskill the co-creators. This workshop will promote ownership of the process by the co-creators, encouraging their engagement in high quality discussions, creativity and innovation when co-creating solutions. Objectives will be formed and an iterative approach will be undertaken such that the content of each workshop will build on the previous one. Co-creators will be asked to brainstorm strategies (e.g. through discussion with family/friends, observation of the media/internet, self-reflection of own experiences), and data prompting techniques will be used to facilitate this process (e.g. showing pictures of people with COPD when hospitalised, or when interfacing with primary care practitioners and participants will be asked ‘how here could this patient advocate to get a referral to PR?’, or ‘would you expect a person with COPD to think about exercise during their admission?’). Co-creators will also be asked to complete fieldwork tasks to gain further understanding to external factors and barriers (e.g. search for stories that include people with COPD in the media, discuss opinions with family or peers). Strategies will be developed, field tested and implemented during this phase (see Table 2).

**Table 2:** Phase 3 and 4 participatory workshop content and fieldwork tasks

Workshop content	Fieldwork tasks
Initial workshop: obtain written informed consent and establish guiding principles of participatory workshops. Upskill co-creators by presenting data obtained during Phases 1 and 2. Develop objectives for Phase 3.	Reflect and diarise the extent to which the determinants of target behaviours are similar or different to their own experiences.
Subsequent workshops: review diaries and reflections of co-creators, use data-prompting and probing questions to seek strategies linked to intervention functions and policy level approaches that align with the determinants of target behaviours. Consider various perspectives. Consider strategies for target behaviours in those hospitalised with an exacerbation of COPD separately from those who visit Respiratory Medicine clinics with ‘stable’ disease. Consider likely challenges and strategies to overcome them.	Seek information and collate ideas regarding strategies to optimise target behaviours. Draw from sources such as peer group discussions, opinions of family and friends, media and the internet. Co-created strategies to be field tested in real time with an opportunity to reflect on successes and challenges, and fine tune them as required.
Final workshops: share final intervention strategies; modify (if necessary) to ensure the findings are representative of the co-creators’ opinions and experiences.	

*Abbreviation*: *COPD* chronic obstructive pulmonary disease

### Phase 4: evaluating the influence of co-created strategies on target behaviours pertaining to PR implementation

The aim of Phase 4 is to evaluate the influence of co-created strategies on target behaviours pertaining to PR implementation at the three tertiary hospitals. With the co-created strategies in place, using the methodology described for Phase 1, target behaviours of referral to PR, attendance to initial appointment, commencement of PR, adherence to PR and provision of a maintenance strategy on PR completion will be re-quantified at each site. At the end of Phase 4, workshop participants (i.e. the co-creators) will re-convene. During this workshop, the influence of co-created strategies on target behaviours will be shared and feedback will be sought to ensure the strategies are representative of co-creators opinions and intended purpose [38, 39].

### Partnering with consumers and end-users

This study aligns with the National Health and Medical Research Council (NHMRC) focus on consumer-driven research [40]. The co-creation research approach ensures meaningful engagement of people with COPD (and their primary support person as able) as ‘co-researchers’, empowering them with an equal participatory footing as healthcare professionals. People with COPD who participate in Phases 3 and 4 workshops will be remunerated for their involvement, demonstrating that their involvement is not tokenistic and we value their input as experts in their own lives. People with COPD will be encouraged to offer opinions which may differ from those of researchers or healthcare professionals. This study also considers the influence of the relationship between people with COPD and those individuals with whom they interact most frequently (i.e. their primary support person). Understanding the dynamics of these relationships is important when attempting to change behaviour, and is possible using a consumer-driven approach.

### Sample size

#### Phase 1 and 4

Using data from the 2018 calendar year from one of the included sites (RPH), there were 1357 cases discharged with a diagnosis of an exacerbation of COPD. Assuming similar numbers across all three sites, over a 5 month period, more than 1000 people will be admitted for an exacerbation of COPD. There are likely to be a similar number of people seen in Respiratory Medicine clinics for COPD over the same time period. Therefore, even following application of exclusion criteria, we anticipate access to a large sample (approximately 2000 participants, across all three sites for both Phase 1 and Phase 4) for analysis.

#### Phase 2 and 3

Regarding Phase 2, a sample will be recruited to reach balance between a sample that is too large (which presents feasibility issues for a detailed analysis) and too small (which is unlikely to achieve rich, contextual appreciation of the interview questions). It is estimated that 15 healthcare professionals and 15 people with COPD (with or without their primary support person) will be recruited to Phase 2. Similarly, regarding Phase 3, an adequate sample size will be recruited to encourage collaboration between healthcare professionals and people with COPD, without being too large which may hinder participation of the co-creators. It is estimated that 10 to 12 participants will be recruited to Phase 3 as co-creators.

### Data analysis

#### Phase 1 and 4

Measurement of target behaviours collected during Phases 1 and 4 will be analysed as an interrupted time series with segmented regression [41]. This design involves collecting data at multiple time points (e.g. monthly for 5 months) before and after a change in practice and represents a robust method to quantify change. In contrast to a simple ‘before-after’ design, multiple measurements is considered a stronger design as it allows for natural time trends and variability to be considered [41].

#### Phase 2

Analysis will follow Braun and Clarke’s six stages of thematic analysis [42] which involves identifying excerpts of data, dividing data into units of code, using and continually adapting the coding frame, building codes into categories and identifying themes [43]. This process will follow investigator triangulation whereby one author (SH) will carry out data analysis, and will be examined by other members of the research team to ensure trustworthiness of the findings. Content will be analysed using an abductive approach that allows the interviewees’ words to drive representations inductively, yet explores the degree of integration with regard to dimensions of capability, opportunity, and motivation [36].

#### Phase 3

There is no data analysis component for Phase 3. A formal process evaluation will be undertaken, reporting the experiences of undertaking participatory, consumer-driven research. For the co-creation process to be clearly understood and reproducible, intervention strategies will be described in accordance with a checklist for reporting intervention co-creation (see Table 3) [38].

**Table 3:** Checklist for intervention co-creation adapted from Leask et al., 2019 [38]

Section	Checklist item
*Planning*	
How was the aim of the study framed?	1. Use each element of the **PRODUCES** framework (**Pr**oblem, **O**bjective, **D**esign, (end)-**U**sers, **C**o-creators, **E**valuation, **S**calability).
Explain the sampling procedure	2. Explain the criteria used for sampling. 3. In what setting did the sampling occur? 4. How many individuals engage as co-creators? (Academic / non-academic stakeholders?) . 5. Describe the co-creators (demographics / groups / other characteristics of interest).
*Conducting*	
How was ownership manifested?	6) Explain the methods used to manifest ownership among the co-creators.
Procedure components	7) What level of participation was there from the co-creators? 8) How was the overall aim presented? 9) How was the purpose of each meeting presented? 10) What were the rules and responsibilities of participation agreed upon?
Procedure methods	11) In which areas did the co-creators require upskilling? 12) What previous evidence was reviewed, and how? 12) If a prototype was developed, describe the prototype and the prototyping process. 14) Describe the frequency and duration of meetings. 15) Give examples of interactive techniques or methods used. 16) Give examples of fieldwork techniques or methods used. 17) Give examples of how iteration occurred during the process.
*Evaluation*	
Process	18) Explain how co-creators satisfaction and contribution was evaluated (e.g. reporting attendance rates, feedback questionnaires, interviews, etc.). 19) How are results reported back to stakeholders and the public?
Outcome	20) Explain how the validity of the outcome and process were evaluated (e.g. face validation, member checking, etc.). 22) Explain plans for formal testing of the effectiveness/scalability of the co-created outcome. 21) Explain outcome of the evaluation (if tested).

### Economic analysis

The burden of COPD to the individual, their family and to the healthcare system is substantial [6]. In addition to improvement in health outcomes, PR has the potential to mitigate some of the healthcare burden associated with COPD. For example, randomised controlled trial data from a study undertaken in the UK demonstrated the relationship between PR and reduction in health service utilisation [44]. During the 12 month follow-up period, although there was no statistically significant between-group difference in the number of people admitted to hospital, the length of stay for those who have attended PR was considerably shorter (10.4 days versus 21.0 days, *p* = 0.022). Alternatively, data from WA [45] from one of the included sites (SCGH) suggests that PR yields reduction in both admissions and hospital length of stay. Based on a sample of 256 people at SCGH, the authors identified a 46% reduction in the number of people admitted to hospital with a COPD exacerbation, and a 62% reduction in total bed days. Further, the association between PR and reduced respiratory-related hospital admissions has been supported by two systematic reviews [11, 12].

This project aims to optimise target behaviours pertaining to the implementation of PR in people with COPD living in Perth, WA. In addition to clinical influence, the financial influence of strategies which seek to optimise the implementation of PR is potentially large. In this project, we will collect information about the cost associated with delivery of the co-created strategies and subsequent PR. We will then use routinely collected data to explore the number of exacerbations and total subsequent length of stay, which will be costed using WA-specific per day costs. An interrupted time series analysis will be used to quantify the change in target behaviours with co-created strategies in place. We will model the effect of these changes on implementation of PR, which in turn will allow us to model the likely cost implications of the strategies used to improve implementation. It is likely that the interventions to optimise target behaviours pertaining to PR implementation will be at either a low cost or free, and may include prompts to physicians and general practitioners to encourage timely referrals, development of infographics or letters to patients to encourage uptake of the service. Thus, the key costs are the cost of the program itself, and the cost offsets are associated with reduced healthcare utilisation [44, 45]. Once subsequent years of cost savings are considered, the net cost of improved implementation of PR is likely to be negative (i.e. cost-saving). Thus, any intervention which, at a negligible cost, improves implementation of PR in the at-risk population is likely to be cost-saving to the WA Health system.

## Discussion

The health benefits associated with PR for people with COPD who experience dyspnoea during daily life are supported by a large evidence base [9]. Earlier work has highlighted problems with implementation and underutilisation of PR [13, 14], and there is limited guidance on how to improve this problem within clinical practice. This study will quantify current clinical practice around implementation of PR in people with COPD living in Perth, WA. Perspectives of healthcare professionals, people with COPD and their primary support person will be sought to gain a deeper understanding to the determinants of target behaviour pertaining to the implementation of PR. Knowledge gained in Phases 1 and 2 will be used as a platform for consumer-driven research. By collaborating with healthcare professionals and people with COPD as co-researchers, their insights and experiences will drive co-creation of strategies which can optimise the implementation of PR in people with COPD. The co-created interventions, setting and target population of this study will be clearly defined to enhance applicability and reproducibility of interventions in clinical practice. Optimising the implementation of PR among people with COPD ensures more people can derive health benefits associated with PR. This research represents a step in the right direction of tackling a global issue. The research process itself will serve as an exemplar for future co-designed research in chronic health. Given that PR has been associated with reduced healthcare utilisation, by improving referral, attendance and adherence to PR there is a potential to mitigate healthcare costs and this will be clarified by our economic analysis.

## Data Availability

The datasets used and/or analysed during the current study are available from the corresponding author on reasonable request.

## Abbreviations

ATS: American Thoracic Society
COPD: Chronic obstructive pulmonary disease
ERS: European Respiratory Society
FSH: Fiona Stanley Hospital
NHMRC: National Health and Medical Research Council
PR: Pulmonary rehabilitation
REDCap: Research Electronic Data Capture
RPH: Royal Perth Hospital
SCGH: Sir Charles Gairdner Hospital
WA: Western Australia

## References

[1] The Global Initiative for Chronic Obstructive Lung Diseases (GOLD) (2020). Global Strategy for Diagnosis, Management and Prevention of COPD: 2020 report.

[2] Soriano JB, Kendrick PJ, Paulson KR, Gupta V, Abrams EM, Adedoyin RA (2020). Prevalence and attributable health burden of chronic respiratory diseases, 1990-2017: a systematic analysis for the global burden of disease study 2017. Lancet Respir Med.

[3] Toelle BG, Xuan W, Bird TE, Abramson MJ, Atkinson DN, Burton DL (2013). Respiratory symptoms and illness in older Australians: the burden of obstructive lung disease (BOLD) study. Med J Aust.

[4] Buist AS, McBurnie MA, Vollmer WM, Gillespie S, Burney P, Mannino DM (2007). International variation in the prevalence of COPD (the BOLD study): a population-based prevalence study. Lancet.

[5] Australian Institute of Health and Welfare (2015). Australian burden of disease study 2015: interactive data on disease burden.

[6] Australian Institute of Health and Welfare (2019). Chronic obstructive pulmonary disease (COPD).

[7] Yang IA, Brown JL, George J, Jenkins S, McDonald CF, McDonald V. The COPD-X Plan: Australian and New Zealand Guidelines for the management of Chronic Obstructive Pulmonary Disease 2020. Australia Lung Foundation/Thoracic Society of Australia and New Zealand. Available at: www.copdx.org.au/guidelines/index.asp. Accessed Sept 2020.

[8] Spruit MA, Singh SJ, Garvey C, ZuWallack R, Nici L, Rochester C (2013). An official American Thoracic Society/European Respiratory Society statement: key concepts and advances in pulmonary rehabilitation. Am J Respir Crit Care Med.

[9] McCarthy B, Casey D, Devane D, Murphy K, Murphy E, Lacasse Y (2015). Pulmonary rehabilitation for chronic obstructive pulmonary disease. Cochrane Database Syst Rev.

[10] Lacasse Y, Cates CJ, McCarthy B, Welsh EJ. This Cochrane Review is closed: deciding what constitutes enough research and where next for pulmonary rehabilitation in COPD. Cochrane Database Syst Rev. 2015;(11):Ed000107. 10.1002/14651858.ED000107 PMC10845864 26593129

[11] Puhan MA, Gimeno-Santos E, Cates CJ, Troosters T (2016). Pulmonary rehabilitation following exacerbations of chronic obstructive pulmonary disease. Cochrane Database Syst Rev.

[12] Moore E, Palmer T, Newson R, Majeed A, Quint JK, Soljak MA (2016). Pulmonary rehabilitation as a mechanism to reduce hospitalizations for acute exacerbations of COPD: a systematic review and meta-analysis. Chest..

[13] Rochester CL, Vogiatzis I, Holland AE, Lareau SC, Marciniuk DD, Puhan MA (2015). An official American Thoracic Society/European Respiratory Society policy statement: enhancing implementation, use, and delivery of pulmonary rehabilitation. Am J Respir Crit Care Med.

[14] Desveaux L, Janaudis-Ferreira T, Goldstein R, Brooks D (2015). An international comparison of pulmonary rehabilitation: a systematic review. COPD.

[15] Cousins JL, Wood-Baker R, Wark PAB, Yang IA, Gibson PG, Hutchinson A (2020). Management of acute COPD exacerbations in Australia: do we follow the guidelines?. ERJ Open Res.

[16] Johnston K, Young M, Grimmer K, Antic R, Frith P (2013). Frequency of referral to and attendance at a pulmonary rehabilitation programme amongst patients admitted to a tertiary hospital with chronic obstructive pulmonary disease. Respirology..

[17] Milner SC, Boruff JT, Beaurepaire C, Ahmed S, Janaudis-Ferreira T (2018). Rate of, and barriers and enablers to, pulmonary rehabilitation referral in COPD: a systematic scoping review. Respir Med.

[18] Johnston KN, Young M, Grimmer KA, Antic R, Frith PA (2013). Barriers to, and facilitators for, referral to pulmonary rehabilitation in COPD patients from the perspective of Australian general practitioners: a qualitative study. Prim Care Respir J.

[19] Cox NS, Oliveira CC, Lahham A, Holland AE (2017). Pulmonary rehabilitation referral and participation are commonly influenced by environment, knowledge, and beliefs about consequences: a systematic review using the theoretical domains framework. J Physiother.

[20] Steiner MC, Roberts CM, Lowe D, Welham S, Searle L, Skipper E (2016). Pulmonary rehabilitation: steps to breathe better. National chronic obstructive pulmonary disease (COPD) audit programme: clinical audit of pulmonary rehabilitation services in England and Wales 2015.

[21] Jones SE, Green SA, Clark AL, Dickson MJ, Nolan A-M, Moloney C (2014). Pulmonary rehabilitation following hospitalisation for acute exacerbation of COPD: referrals, uptake and adherence. Thorax..

[22] Harris D, Hayter M, Allender S (2008). Improving the uptake of pulmonary rehabilitation in patients with COPD: qualitative study of experiences and attitudes. Br J Gen Pract.

[23] Keating A, Lee AL, Holland AE (2011). Lack of perceived benefit and inadequate transport influence uptake and completion of pulmonary rehabilitation in people with chronic obstructive pulmonary disease: a qualitative study. J Physiother.

[24] Sabit R, Griffiths TL, Watkins AJ, Evans W, Bolton CE, Shale DJ (2008). Predictors of poor attendance at an outpatient pulmonary rehabilitation programme. Respir Med.

[25] Harrison SL, Robertson N, Apps L, Steiner MC, Morgan MDL, Singh SJ (2015). “We are not worthy” – understanding why patients decline pulmonary rehabilitation following an acute exacerbation of COPD. Disabil Rehabil.

[26] Osadnik C, Gordon C, Gerstman E (2019). Referrals to pulmonary rehabilitation after acute exacerbations of COPD: a mixed-methods evaluation. Eur Respir J.

[27] McNaughton A, Weatherall M, Williams G, Delacey D, George C, Beasley R (2016). An audit of pulmonary rehabilitation program. Clin Audit.

[28] Spitzer KA, Stefan MS, Priya A, Pack QR, Pekow PS, Lagu T (2019). Participation in pulmonary rehabilitation after hospitalization for chronic obstructive pulmonary disease among Medicare beneficiaries. Ann Am Thorac Soc.

[29] Sayer A. Realism and social science. London: SAGE publications; 2000.

[30] Bhaskar R. A realist theory of science. 2nd ed. London: Routledge; 2008.

[31] Wiltshire G (2018). A case for critical realism in the pursuit of interdisciplinarity and impact. Qual Res Sport Exer Health.

[32] Wilson R (2014). The Routledge companion to accounting education.

[33] Maxwell J, Smith RJ (2013). A realist approach for qualitative research.

[34] Harris PA, Taylor R, Thielke R, Payne J, Gonzalez N, Conde JG (2009). Research electronic data capture (REDCap)—a metadata-driven methodology and workflow process for providing translational research informatics support. J Biomed Inform.

[35] Hug S, Cavalheri V, Gucciardi DF, Hill K. Optimising the implementation of pulmonary rehabilitation in people with COPD. Available at: https://osf.io/qet25/?view_only=e185130575a54e2aa4d95435977dda1a. Accessed 13 Sept 2020. 10.1186/s12890-020-01322-4 PMC7607703 33138804

[36] Michie S, van Stralen MM, West R (2011). The behaviour change wheel: a new method for characterising and designing behaviour change interventions. Implement Sci.

[37] Cane J, O'Connor D, Michie S (2012). Validation of the theoretical domains framework for use in behaviour change and implementation research. Implement Sci.

[38] Leask CF, Sandlund M, Skelton DA, Altenburg TM, Cardon G, Chinapaw MJM (2019). Framework, principles and recommendations for utilising participatory methodologies in the co-creation and evaluation of public health interventions. Res Involv Engagem.

[39] Bergold JTS (2012). Participatory Research Methods: A Methodological Approach in Motion.

[40] National Health and Medical Research Council (2016). Statement on Consumer and Community involvement in Health and Medical Research, Consumers Health Forum of Australia.

[41] Bernal JL, Cummins S, Gasparrini A (2017). Interrupted time series regression for the evaluation of public health interventions: a tutorial. Int J Epidemiol.

[42] Braun V, Clarke V, Weate P, Smith B, Sparkes AC (2016). Using thematic analysis in sport and exercise research. Routledge handbook of qualitative research in sport and exercise.

[43] Oliver SR, Rees RW, Clarke-Jones L, Milne R, Oakley AR, Gabbay J (2008). A multidimensional conceptual framework for analysing public involvement in health services research. Health Expect.

[44] Griffiths TL, Burr ML, Campbell IA, Lewis-Jenkins V, Mullins J, Shiels K (2000). Results at 1 year of outpatient multidisciplinary pulmonary rehabilitation: a randomised controlled trial. Lancet..

[45] Cecins N, Geelhoed E, Jenkins SC (2008). Reduction in hospitalisation following pulmonary rehabilitation in patients with COPD. Aust Health Rev.

